# Automated Oxygen Delivery in Hospitalized Patients with Acute Respiratory Failure: A Pilot Study

**DOI:** 10.1155/2019/4901049

**Published:** 2019-02-03

**Authors:** Foteini Malli, Stelios Boutlas, Nick Lioufas, Konstantinos I. Gourgoulianis

**Affiliations:** ^1^Respiratory Medicine Department, University of Thessaly, Faculty of Medicine, Larissa, Greece; ^2^Technological Educational Institute of Thessaly, Nursing Department, Larissa, Greece

## Abstract

**Background and Objectives:**

Despite its' proven benefits, oxygen therapy may be complicated with potential adverse events such as hypoxemia or hyperoxia-driven hypercapnia. Automated oxygen delivery systems may aid in avoiding these complications. The scope of the present study is to test the efficacy and safety of a new automated oxygen delivery device.

**Methods:**

This study included 23 patients with acute respiratory failure (ARF) hospitalized in the Respiratory Medicine Department of the University Hospital of Larissa. Both patients with purely hypoxemic or hypercapnic ARF were included. Automated oxygen administration was performed with *Digital Oxygen Therapy*, a new closed-loop system designed to automatically adjust oxygen flow according to target oxygen saturation (SpO_2_) of 88–92% for hypercapnic patients and 92–96% for purely hypoxemic patients with ARF. The device was applied for 4 hours. Arterial blood gas analysis was performed at 1 hour and 3 hours following the device application.

**Results:**

Mean age was 72.91 ± 13.91 years. Twelve patients were male, and 11 were female. The majority of patients suffered from hypercapnic respiratory failure (*n*=13, 56.5%). At 1 hour and 3 hours, SpO_2_ and PaO_2_ displayed excellent correlation (*p* < 0.001, *r* = 0.943, and *p* < 0.001, *r* = 0.954, respectively). We did not observe any adverse events associated with the device.

**Conclusions:**

Our results indicate that automated oxygen treatment is feasible and safe in hospitalized patients with acute respiratory failure. Further studies are required in order to assess the long-term effects of automated oxygen delivery systems.

## 1. Introduction

The benefits of oxygen therapy in the setting of acute respiratory failure (ARF) as well as long-term oxygen therapy (LTOT) in patients with chronic respiratory failure have been well-documented [1, 2]. LTOT has proven benefits in survival, quality of life, and neuropsychological functions and modest benefits in pulmonary haemodynamics [2]. Additionally, emergency oxygen use has documented implications in the survival of patients with ARF [1].

A potential risk of uncontrolled oxygen delivery is hyperoxia-induced hypercapnia especially in patients with chronic obstructive pulmonary disease (COPD), chest wall deformities, or muscle weakness. High concentration oxygen delivered in patients with CO_2_ retention may result in respiratory acidosis mainly due to reduced “hypoxic drive” and ventilation-perfusion mismatch deterioration [2, 3], while hypoxemia is also associated with serious adverse outcomes [4]. In order to avoid these complications, current guidelines suggest monitoring of oxygen delivery to target oxygen saturation (SpO_2_) of 88–92% for hypercapnic patients and 94–98% for normocapnic subjects with acute respiratory failure [1, 2]. However, available constant flow devices do not have the ability to titrate oxygen flow according to patients corresponding SpO_2_, while studies have shown poor compliance of health professional with international guidelines [5].

Previous researchers have examined the efficacy of various automated oxygen delivery devices. Automated oxygen flow titration has been tested during induced hypoxemia in healthy subjects [6] as well as exercise-induced hypoxemia in patients with chronic lung disease [7]. Few studies exist assessing the use of automated oxygen delivery in ARF [8, 9]. Additionally, data in the literature are sparse regarding the efficacy of similar systems in hopsitalized patients. Moreover, the few available data have not adequately addressed the role of automated oxygen delivery systems in hypercapnic patients in which worsening of CO_2_ retention during oxygen treatment may lead to respiratory acidosis.

The scope of the present study is to test the efficacy and safety of a new device (*Digital Oxygen Therapy*) to titrate oxygen flow in real-time acute setting.

## 2. Methods

### 2.1. Subjects

The present prospective cohort study was conducted at the University Hospital of Larissa, Larissa, Greece. Patients were recruited by consecutive sampling from the Respiratory Medicine Department. Patients were eligible if they suffered from ARF of any cause and any degree of severity. Both patients with hypoxemic and hypercapnic respiratory failure were included in the study. Hypoxemic and hypercapnic respiratory failures were defined according to current guidelines [1]. In brief, hypoxemic respiratory failure was defined as PaO_2_ < 60 mm Hg with a normal or low PCO_2_ level in ambient air and hypercapnic respiratory failure as PCO_2_ > 45 mmHg (despite of PaO_2_ levels). All patients with hypercapnic respiratory failure in our study were hypoxemic (PaO_2_ < 60 mmHg) and required oxygen therapy. We excluded patients requiring noninvasive or invasive mechanical ventilation. The study was approved by the University Hospital of Larissa Ethics Committee. All subjects were conscious during the study period and gave verbal and written informed consent to participate in the study.

A detailed medical history was obtained from all subjects. All participants underwent clinical examination and arterial blood gas (ABGs) analysis (model 1630; Instrumentation Laboratories, Milan Italy) both at admission and right before the application of the device. As most patients were under oxygen therapy, we calculated alveolar to arterial (A-a) gradient for the assessment of their oxygenation, by the formula: P_(A−a)_O_2_ = (713 × FiO_2_ − 1.25 × PCO_2_) − PaO_2_. We included patients within the first 24 hours following admission.

### 2.2. Study Design

**Figure 1:**
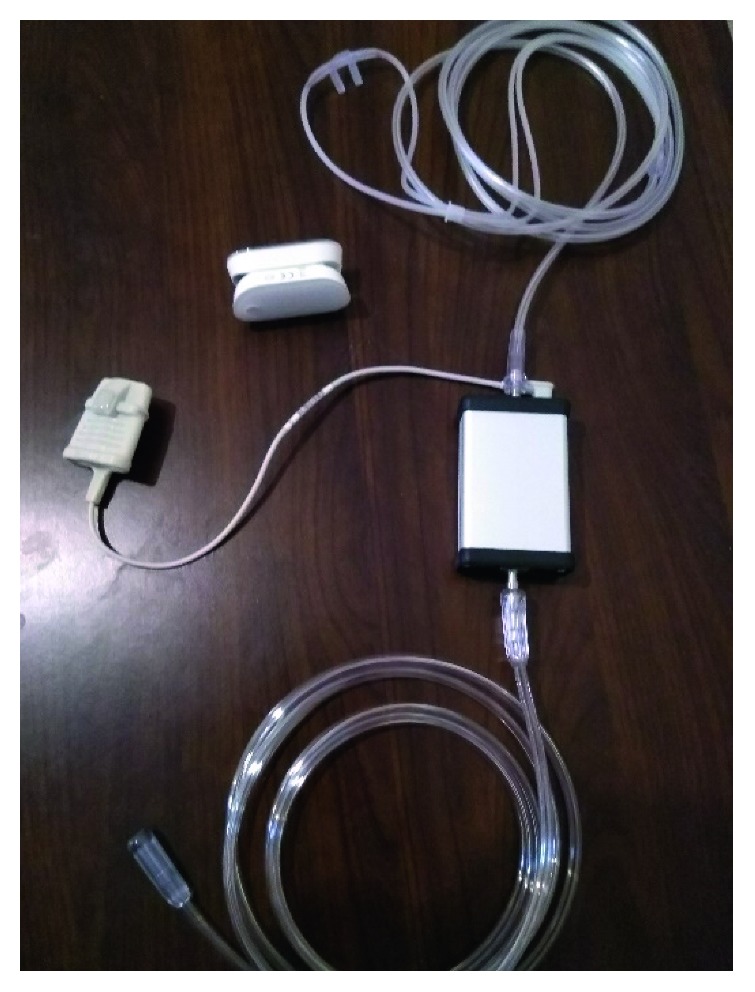
*Digital Oxygen Therapy* device.

Automated oxygen administration was performed with *Digital Oxygen Therapy* (Figure 1). The device was set to maintain a constant SpO_2_ between 88% and 92% for hypercapnic patients and between 92% and 96% for purely hypoxemic subjects. Nasal cannula was used in order to deliver oxygen. *Digital Oxygen Therapy* device was applied for 4 hours in each patient enrolled. During the application of the device, patients were monitored continuously with a pulse oximeter (Nonin Onyx II, model 9560, Nonin Medical, Minnesota, USA) with a finger probe. To further examine the efficacy of the device to maintain a constant SpO_2_, we performed ABGs analysis at regular intervals (specifically at 1 hour and 3 hours of the device application). The primary outcome of the study was SpO_2_ and PaO_2_ correlation at 1 and 3 hr following the application of the device.

### 2.3. Automated Oxygen Delivery Device

The device is a closed-loop system designed to automatically adjust oxygen flow according to the patients' oxygenation. The operation of the oxygen delivery system depends on a microcontroller, an electromagnet valve, and a pulse oximeter. The microcontroller continuously receives the data from the oximeter that corresponds to the patients SpO_2_ and increases or decreases the flow according to the SpO_2_ variations. According to the information that the device has received, it fluctuates oxygen flow through the electromagnet valve until the desired SpO_2_ is reached. If the SpO_2_ is higher than the preset target, it closes the valve in order to reduce flow (0.5 l change over 2 seconds), and if the SpO_2_ is lower than the target, it opens the valve to increase oxygen flow (2 L change over 5 seconds). The device weighs 870 grams, and the algorithm of function uses C++ programming language. The main parameter of the algorithm is SpO_2_ which is taken into account at a rate of 1 value per 3 seconds. A proportional controller (A4 mega 398) is used to adjust flow from 0 to 30 L/min (flow accuracy ±0.1 L/min). The device has an audio alarm that is set by the operating clinician in predefined values of SpO_2_ in order to avoid hyperoxemia and hypoxemia. The device is able to collect and store SpO_2_ and flow data and has an installed Wi-Fi technology with the future ability to install Bluetooth technology. *Digital Oxygen Therapy* is portable, has an installed rechargeable battery, and is patented and marketed. The dimensions of the device are as follows: 8.5 × 5.5 × 3.5 cm. The operating temperature varies from −30°C to +50°C.

### 2.4. Statistical Analysis

Data are presented as mean ± SD unless otherwise indicated. Categorical variables are presented as percentages unless otherwise indicated. Normal distribution was assessed by the Kolmogorov–Smirnov test. Univariate correlations were performed by Pearson's correlation coefficient or by Spearman's correlation coefficient according to variable distribution. A *p* value of <0.05 was considered to be statistically significant. Statistical analysis and graphics were performed using the SPSS 16 statistical package (SPSS Chicago, IL).

## 3. Results

The study population consisted of 23 patients with ARF. Mean age was 72.91 ± 13.91 years (Table 1). Of the patients studied, 12 were male and 11 were women (Table 1). The patients were included in the study 4.17 ± 3.77 hours following admission. The respiratory rate at admission was 26.86 ± 4.29 breaths/minute. Mean SpO_2_ at admission (while breathing room air) was 84.3 ± 4.96%, mean PaO_2_ at admission was 52.73 ± 7.65 mmHg, mean PCO_2_ was 48.48 ± 17.33 mmHg, mean pH was 7.41 ± 0.71 (range 7.19–7.51), and mean P_(A-a)_O_2_ was 99.86 ± 132.38 mmHg. Table 1 presents PaO_2_, PCO_2_, and pH just before the application of the device. Most patients suffered from acute exacerbation of COPD (*n*=8), while 7 patients suffered from pneumonia, 3 cases had acute exacerbation of asthma, 3 subjects had exacerbation of bronchiectasis, and 2 patients were diagnosed with pulmonary embolism. The majority of patients had hypercapnic respiratory failure (*n*=13, 56.5%). Three patients had a background history of obesity hypoventilation syndrome.

**Figure 2:**
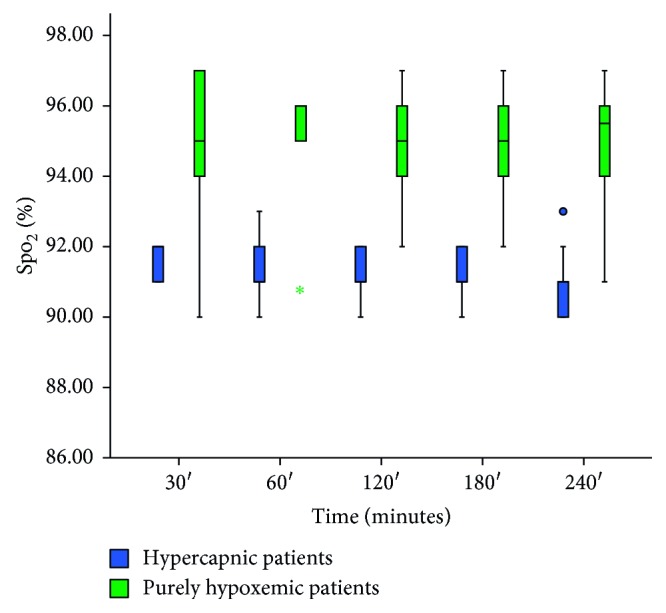
Oxygen saturation (SpO_2_) of hypoxemic (green boxes) and hypercapnic (blue boxes) patients at various intervals during the application of the device.

**Figure 3:**
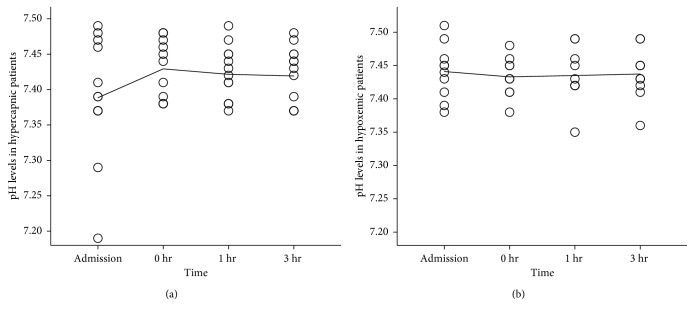
pH levels of hypercapnic and purely hypoxemic ARF patients at admission, just before the application of the device (0 hr), 1 hour after the application of the device (1 hr), and 3 hours after the application of the device (3 hr). The line corresponds to the mean value of each group.

**Figure 4:**
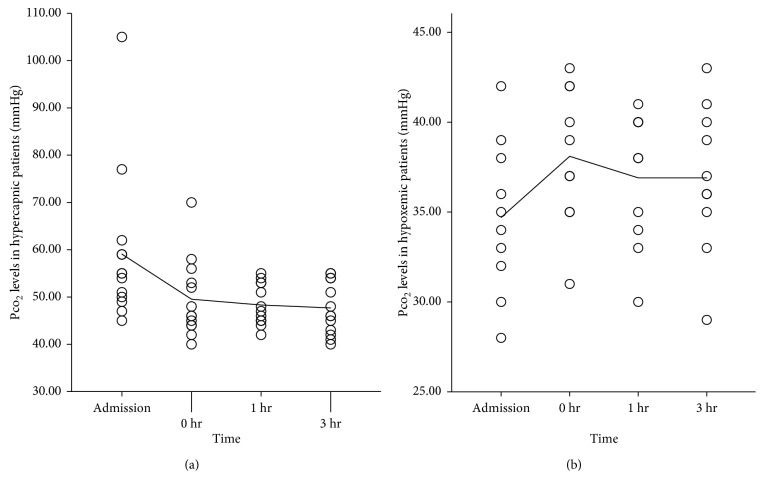
PCO_2_ levels of hypercapnic and purely hypoxemic ARF patients at admission, just before the application of the device (0 hr), 1 hour after the application of the device (1 hr), and 3 hours after the application of the device (3 hr). The line corresponds to the mean value of each group.

**Figure 5:**
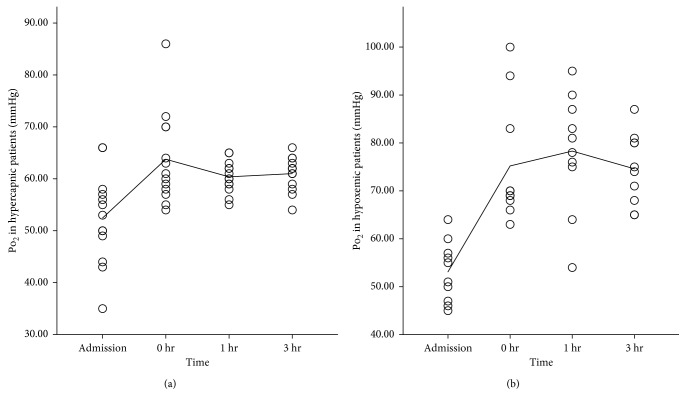
pH levels of hypercapnic and purely hypoxemic ARF patients at admission, just before the application of the device (0 hr), 1 hour after the application of the device (1 hr), and 3 hours after the application of the device (3 hr). The line corresponds to the mean value of each group.

**Figure 6:**
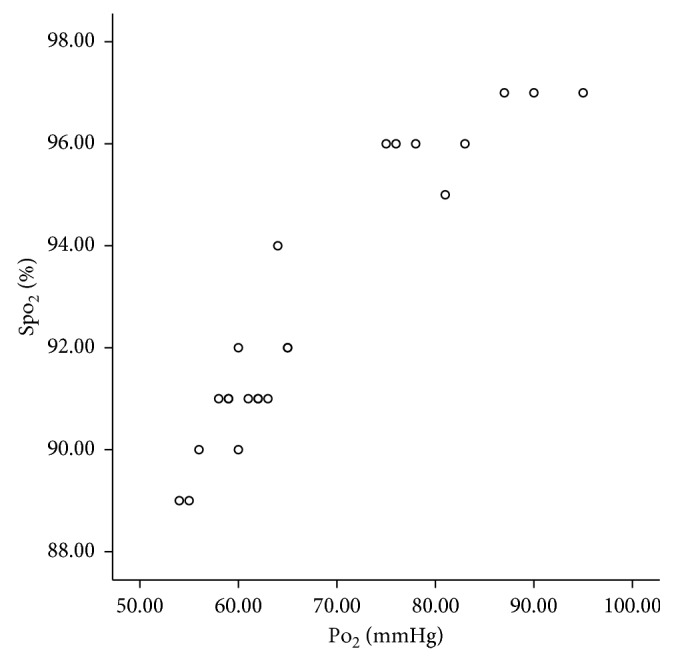
Association of partial arterial oxygen pressure (PO_2_) with oxygen saturation (SpO_2_) at 60 minutes (*p* < 0.001, *r* = 0.943).

Figure 2 presents the SpO_2_ of both hypercapnic and purely hypoxemic patients during the study period. A typical variation of SpO_2_ and oxygen flow in a study subject with purely hypoxemic ARF and a typical recording curve of a patient with purely hypoxemic ARF are presented at Supplementary Figures S1 and S2, respectively, in Supplementary Materials provided online. To further examine the efficacy of the device to maintain a constant SpO_2_, we performed ABGs analysis at 1 hour and 3 hours following the device application. Mean PCO_2_ was 43.34 ± 7.02 mmHg (range 30–55 mmHg), mean pH was 7.42 ± 0.3 (range 7.35–7.49) at 1 hour, mean PCO_2_ was 43.00 ± 7.31 mmHg (range 29–55 mmHg), and mean pH was 7.42 ± 0.4 (range 7.36–7.49) at 3 hours following the application of the device. Figures 3, 4, and 5 display pH, PCO_2_, and PaO_2_ change over time (respectively) in both hypercapnic and hypoxemic ARF. For a graphic presentation of ABGs at admission, just before the application of the device, at 1 hour and 3 hours following the application of the device, refer to Supplementary Figures S3, S4, S5, and S6, respectively, in Supplementary Materials available online. The two variables, SpO_2_ and PaO_2_, displayed excellent correlation in both time frames, i.e., 1 hour and 3 hours (*p* < 0.001, *r* = 0.943, and *p* < 0.001, *r* = 0.954, respectively, Figure 6). Additionally, we tested manually the ability of the device to maintain SpO_2_ in the predefined range at regular intervals (i.e., at 30, 60, 90, 120, 180, and 240 minutes following the device application). For a graphic presentation of SpO_2_ at different time intervals, refer Figure S7 in Supplementary Materials available online. Of the SpO_2_ data recorded per patient, none fell outside the target range for more than 1 minute. SpO_2_ was within the target range for 91.69% (±1.31) of time recorded for hypercapnic patients and 91.39% (±1.00) of time recorded for hypoxemic patients. Hypercapnic patients presented 7.07 % (±1.18) of time with SpO_2_ ≥ 93%, and hypoxemic patients presented 2.60% (±1.39) of time with SpO_2_ ≤ 87%. We did not observe any adverse events associated with the device. We did not observe any problems with the signaling of the pulse oximeter (i.e., due to low perfusion). The oxygen mask was adequate for the device, and we did not experience any problems with it. None of the patients presented hyperoxia-induced hypercapnia.

## 4. Discussion

In the present study, we have tested the efficacy of a new automated oxygen delivery device in hospitalized patients with acute respiratory failure. Importantly, we have included both patients with purely hypoxemic or hypercapnic respiratory failure in real-time acute settings. Additionally, we have tested the efficacy of the device with arterial blood gases, and we observed that oxygen saturation (as measured by the oximeter) and partial arterial oxygen pressure (as tested by arterial blood gas analysis) show excellent correlation. Our results provide evidence that automated oxygen delivery with *Digital Oxygen Therapy* is both feasible and safe. Although our data are not sufficient to conclude that our device is better than standard practice or similar devices [6, 7, 9, 10], our findings provide further support that automated oxygen delivery systems may be an effective alternative to constant oxygen flow systems.

Current clinical practice uses oxygen flow meters with constant flow that requires manual adjustments in order to maintain SpO_2_ in the target range. We have developed a closed-loop system that continuously adjusts oxygen flow according to the patients' SpO_2_ that is predefined by the clinician. In the present study, we have evaluated the efficacy of the device in real-time settings and have found that the device is effective in maintaining SpO_2_ in the target zone. Others have previously tested the ability of automated oxygen therapy systems to adjust oxygen supply according to the patients' needs [6, 7, 10]. Lelouche et al. [6] have assessed the effectiveness of an automated oxygen delivery device (FreeO_2_, Oxynov, Quebec, Canada) in induced hypoxemia in healthy subjects and reported that the application of the device was associated with fewer rates of severe hypoxemia and more time within the SpO_2_ target. In another study by Cirio et al. [7], a similar closed-loop system (O_2_ Flow regulator, Dima, Italy) was applied in chronic lung disease patients with exercise-induced desaturation with encouraging results in terms of better oxygenation and reduced workload. In the same context, Rice et al. [10] applied a similar device (AccuO2, Optisat medical, Mineapolis, MN) in chronic COPD patients that resulted in maintenance of SpO_2_ closer to the target range and higher conservation time of a given O_2_ supply. Recently, L'Her et al [9] demonstrated that automated oxygen administration in the emergency department results in higher time spent within the SpO_2_ range, lower time with hyperoxia and hypoxemia, and better weaning from oxygen delivery. We have assessed the efficacy of a similar closed-loop system (*Digital Oxygen Therapy*) in the acute setting of real patients with various diseases including COPD and bronchiectasis, and most importantly, we have included in the analysis hypercapnic patients who may benefit the most by a SpO_2_ driven device. We have tested the efficacy of the device with ABGs measured during the application of *Digital Oxygen Therapy* and found excellent correlation with PO_2_, providing further support for the effectiveness of the device. The different closed-loop systems [6, 7, 10] share some similarities but have different technical parameters such as reaction time to SpO_2_, flow accuracy, flow range, and availability of the alarm. Some of the most important differences in technical parameters of our device with the ones previously published may be the response time to SpO_2_, the rate that the algorithm takes SpO_2_ into account and the flow range. However, one should be reserved before definite conclusions can be drawn since comparative studies of the available devices are not available.

Oxygen therapy is essential in the treatment of patients with respiratory failure. However, studies have observed poor compliance of health professionals with international guidelines concerning the use of oxygen therapy in the acute setting [5]. Potential risks associated with the available oxygen delivery systems are hypoxemia or hyperoxia-induced hypercapnia. Studies have shown that high flow oxygen is associated with increased mortality in COPD patients [3, 4, 11]. Optimizing oxygen delivery may have the potential to decrease morbidity associated with COPD along with minimizing hyperoxia-associated risks [12]. Additionally, by providing automated titration of oxygen delivery in a predefined target range may reduce the workload of the medical staff provided fail-safe mechanisms exist. Studies have reported the deleterious effects of uncontrolled oxygen therapy in patients with increased risk of hyperoxia-induced hypercapnia in various conditions [4,13–15]. Automated oxygen systems may aid in avoiding these complications.

Although closed-loop oxygen delivery systems may have a broad clinical potential [16], certain issues need to be addressed before one discusses its' wide applications in patients with respiratory failure. Researchers have underlined the need for close auditing since the available devices do not alert the clinician of increasing supplemental oxygen requirements or may suffer defects and therefore behave inappropriately in controlling SpO_2_ [17]. We observed no adverse event suggesting that the device is safe. As others have suggested, improvements of physiological sensors may benefit oxygenation control since SpO_2_ driven devices do not take into account other important sources of information that could determine oxygen requirements [18].

Our study has several limitations. We acknowledge that our device was applied for a short period of time and therefore definite conclusions concerning the safety of long-term use of the system cannot be drawn. Additionally, we have included only hospitalized patients with ARF, and thus, our results may not adequately extrapolate in outpatients requiring long-term oxygen therapy. We certainly acknowledge that the number of the patients included in our study is relatively small. However, the aim of the study was to test the efficacy and safety of a newly developed device and not to find a difference between patient groups where a larger group of patients would definitely be needed. Although we did not observe any such problems with the signaling of the pulse oximeter, we acknowledge that the study sample is too low to extract any definite conclusions.

In conclusion, our results suggest that automated oxygen titration with Digital Oxygen Therapy is both feasible and safe in hypoxemic as well as hypercapnic acute respiratory failures. Besides the efficacy of closed-loop systems as described in our study and others [8, 9], further long-term studies with a larger cohort and further parameters included are warranted.

## Figures and Tables

**Table 1:** Clinical characteristics of the patients studied.

Parameter	*N* or mean ± SD
Number of patients	23
Age (years)	72.91 ± 13.91
Gender (M/F)	12/11
PaO_2_ (mmHg)	68.73 ± 11.90
PCO_2_ (mmHg)	44.56 ± 8.73
pH (range)	7.43 ± 0.36 (7.38–7.48)
P_(A-a)_O_2_ (mmHg)	113.34 ± 53.31

Arterial blood gas analysis results correspond to the measurements just before the application of the device.

## Data Availability

The data supporting the conclusions of the present study are presented within the article. The detailed clinical data are not publicly available in order to ensure study subjects anonymity and protect confidentiality. Data are available upon request.

## Abbreviations

M/F: Male/female
PaO_2_: Partial arterial oxygen pressure
PCO_2_: Partial arterial carbon dioxide pressure
P_(A−a)_O_2_: Alveolar to arterial gradient.

## Supplementary Materials

Supplementary Materials A typical variation of SpO_2_ and oxygen flow in a study subject with purely hypoxemic ARF and a typical recording curve of a patient with purely hypoxemic ARF is presented at Supplementary Figures S1 and S2, respectively, in Supplementary Materials provided online. For a graphic presentation of ABGs at admission, just before the application of the device, at 1 hour and 3 hours following the application of the device, refer Supplementary Figures S3, S4, S5, and S6, respectively, in Supplementary Materials available online. For a graphic presentation of SpO_2_ at different time intervals, refer Figure S7 in Supplementary Materials available online.
