# Supplementary material for: Automated Oxygen Delivery in Hospitalized Patients with Acute Respiratory Failure: A Pilot Study
Source: Can Respir J. 2019 Feb 3;2019:4901049. doi: 10.1155/2019/4901049 (PMC6377968; doi:10.1155/2019/4901049)

Figure S1. Typical variations of Oxygen saturation (S_{pO2}) (blue line) and the corresponding oxygen flow (green line) in a patient with purely hypoxemic acute respiratory failure (ARF).

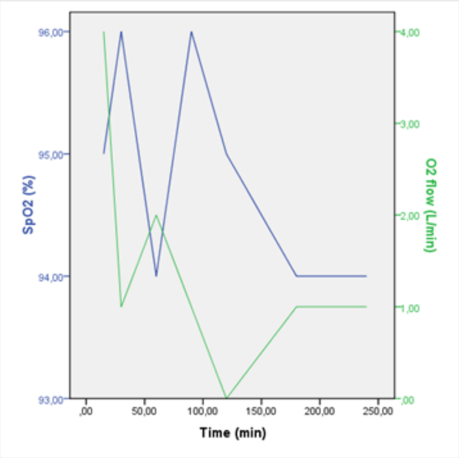

Figure S2. Typical recording curves of a patient with purely hypoxemic respiratory failure presenting variations of oxygen flow (lower panel) according to patients Oxygen saturation (S_{pO2}) (upper panel) over a 30-minute period.

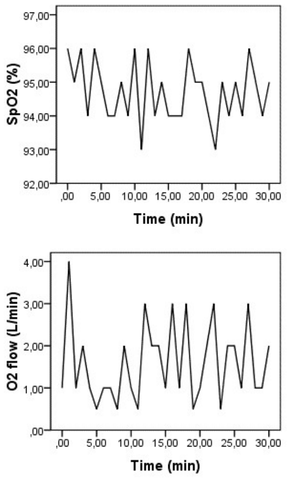

Figure S3. Arterial blood gas (ABGs) analysis of patients at admission. The blue dots correspond to P_{O2} and P_{CO2} values and the green dots correspond to pH values.

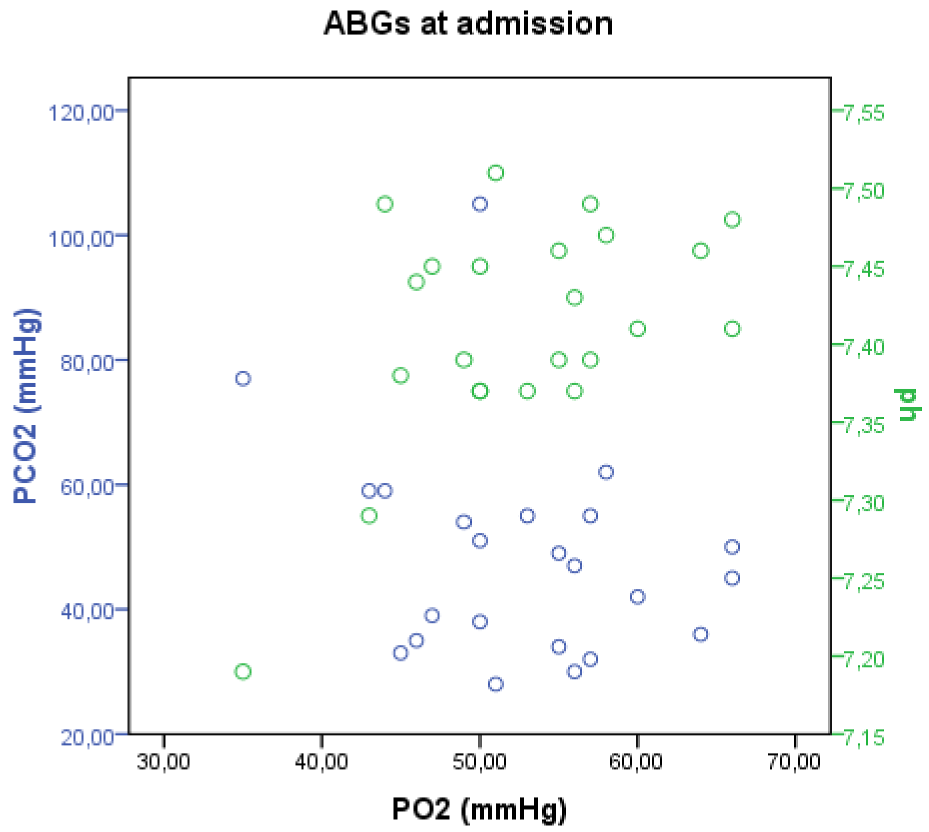

Figure S4. Arterial blood gas (ABGs) analysis of patients just before the application of the device. The blue dots correspond to P_{O2} and P_{CO2} values and the green dots correspond to pH values.

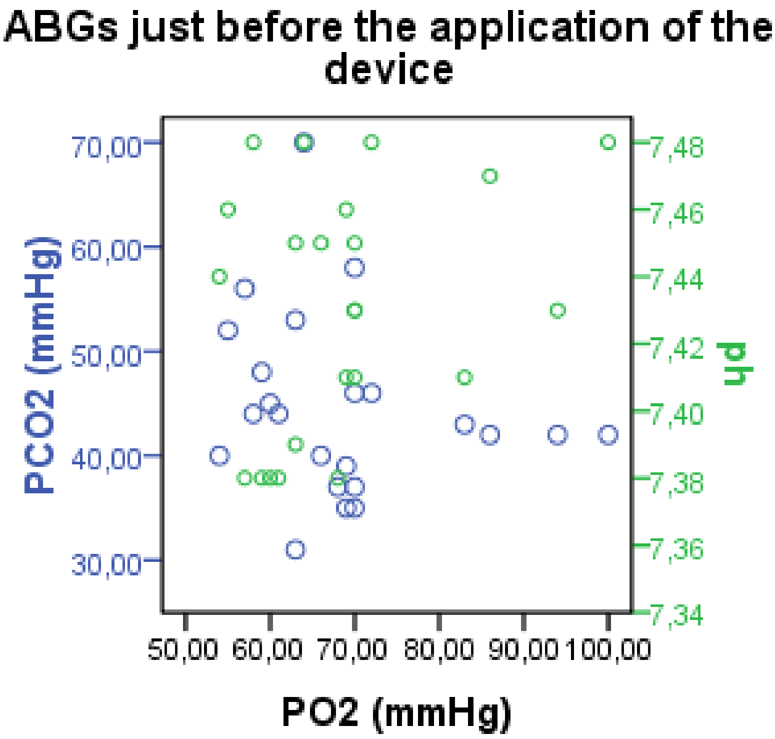

Figure S5. Arterial blood gas (ABGs) analysis of patients at 1 hour following the application of the device. The blue dots correspond to P_{O2} and P_{CO2} values and the green dots correspond to pH values.

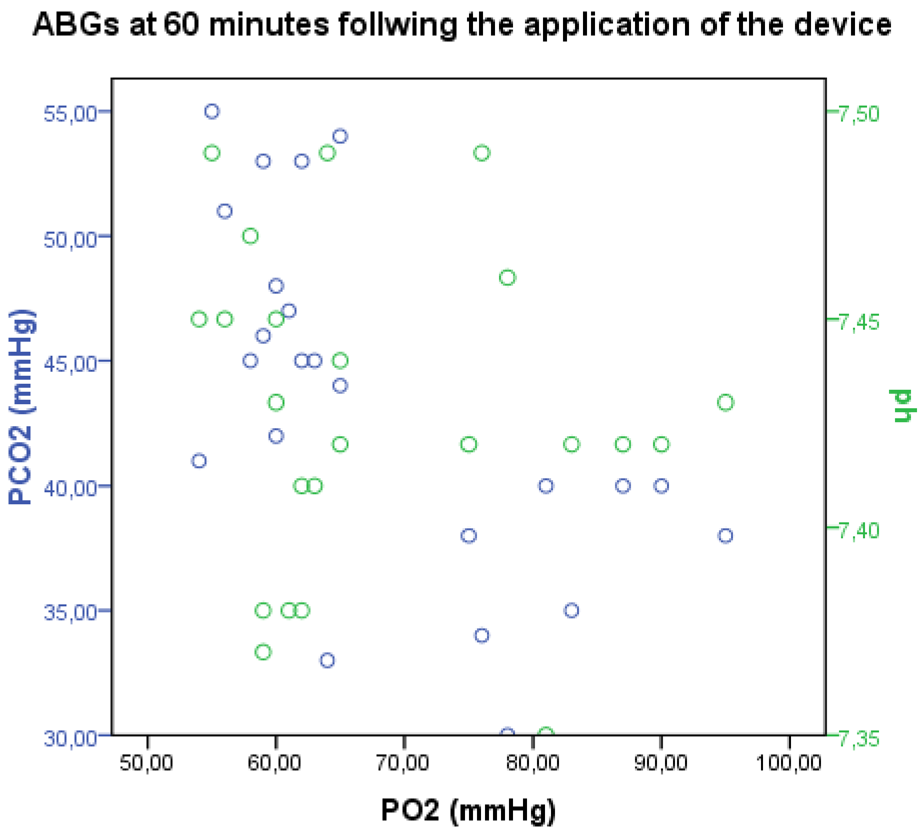

Figure S6. Arterial blood gas (ABGs) analysis of patients at 3 hours following the application of the device. The blue dots correspond to P_{O2} and P_{CO2} values and the green dots correspond to pH values.

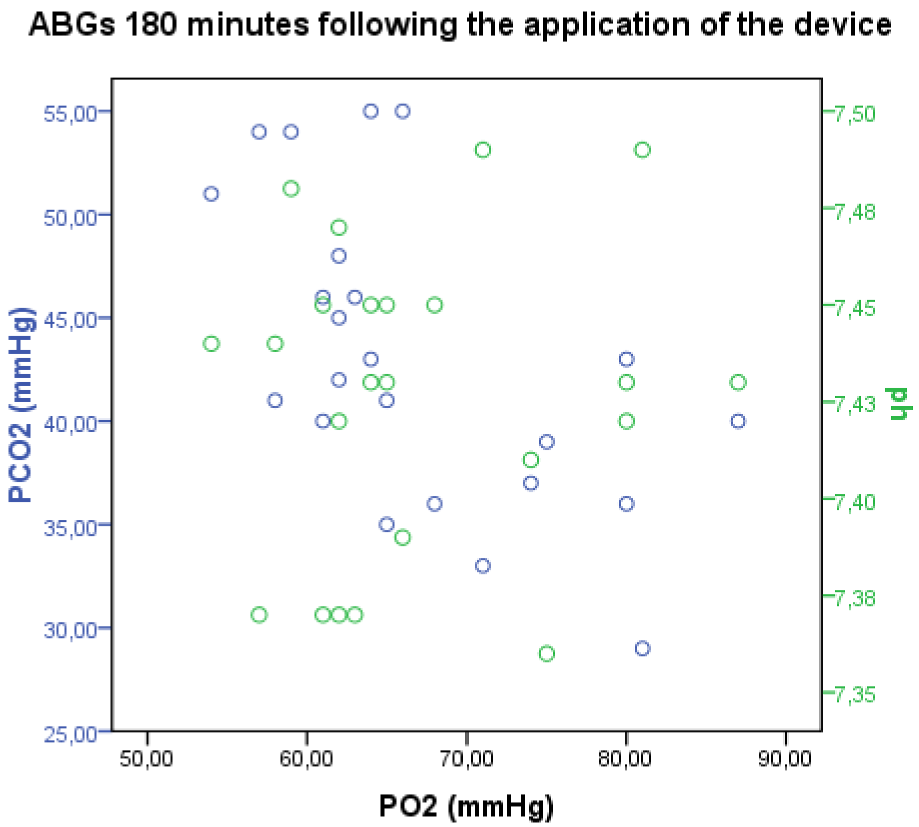

Figure S7. S_{pO2} at different time points (i.e. 15, 30, 60, 120, 180, 240 minutes) following the application of the device in hypercapnic and hypoxemic patients. The number of dots is fewer than the patients studied since some patients displayed the same values (in that case the dots appear darker). The line corresponds to the mean value of each group.

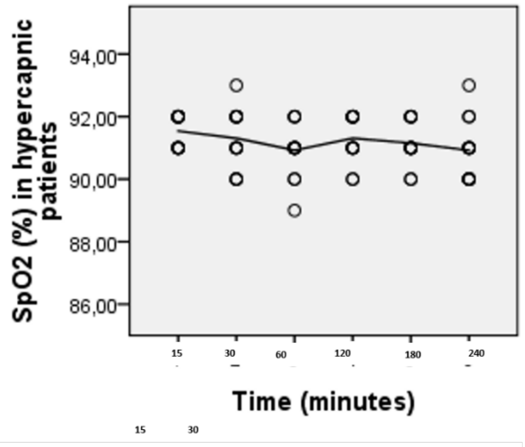

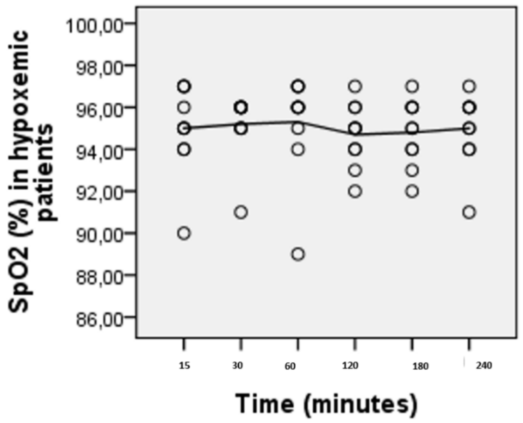

Supplement: Supplementary Materials — A typical variation of SpO2 and oxygen flow in a study subject with purely hypoxemic ARF and a typical recording curve of a patient with purely hypoxemic ARF is presented at Supplementary Figures S1 and S2, respectively, in Supplementary Materials provided online. For a graphic presentation of ABGs at admission, just before the application of the device, at 1 hour and 3 hours following the application of the device, refer Supplementary Figures S3, S4, S5, and S6, respectively, in Supplementary Materials available online. For a graphic presentation of SpO2 at different time intervals, refer Figure S7 in Supplementary Materials available online. [file 4901049.f1.pdf]
